# MRI-T2^*^ quantification of cardiac iron content correlates with extramedullary hematopoiesis and homozygous β0 genotypes in transfusion-dependent thalassemia

**DOI:** 10.3389/fmed.2025.1627902

**Published:** 2025-11-18

**Authors:** Siyu Tan, Xiaojing Ning, Chunxia Zhu, Guowei Chen, Rong Kong, Yugui Huang, Cheng Tang, Cunan Liu, Peng Peng

**Affiliations:** 1 Department of Radiology, The First Affiliated Hospital of Guangxi Medical University, Nanning, Guangxi Zhuang Autonomous Region, China; 2 NHC Key Laboratory of Thalassemia Medicine, Nanning, Guangxi Zhuang Autonomous Region, China

**Keywords:** transfusion-dependent thalassemia, extramedullary hematopoiesis masses, cardiac T2^*^, homozygous β0 genotypes, magnetic resonance imaging

## Abstract

**Aim:**

This study examined the correlation between cardiac iron content and extramedullary hematopoiesis (EMH) masses, and evaluated the predictive value of cardiac iron content for homozygous β0 genotypes in transfusion-dependent thalassemia (TDT) patients.

**Methods:**

This prospective study enrolled 77 thalassemia patients from October 2022 to June 2024. MRI was used to quantify EMH mass number (N_EMH_) and maximum diameter (D_EMH_), as well as cardiac and liver T2^*^ values via T2^*^ mapping. Clinical data were collected from hospital records. Participants were grouped by transfusion dependence, N_EMH_, D_EMH_, and genotype. Multiple linear regression and receiver operating characteristic (ROC) analyses were applied to identify cardiac T2^*^ predictors and the discriminative power of cardiac T2^*^ for homozygous β0 genotypes in transfusion-dependent thalassemia (TDT) patients.

**Results:**

Of 77 patients, 58 had TDT and 19 had NTDT. Among TDT patients, 42 were EMH (+) and 16 EMH (–). The EMH (+) group was subdivided by N_EMH_ into 0 < N_EMH_ < 10 (*n* = 13) and N_EMH_ ≥ 10 (*n* = 29), and by the median D_EMH_ into 0 < D_EMH_ < 2.85 cm (*n* = 21) and D_EMH_ ≥ 2.85 cm (*n* = 21). Genotyping was performed on 25 β-TDT patients, identifying 14 as homozygous β0 and 11 as heterozygous β0β+. The TDT patient group showed a significantly higher prevalence of EMH than the NTDT group (*p* = 0.001). In TDT patients, the N_EMH_ ≥ 10 or D_EMH_ ≥ 2.85 cm group had elevated cardiac T2^*^ (*p* = 0.018; *p* = 0.014). D_EMH_ ≥ 2.85 cm remained an independent predictor of cardiac T2^*^ (*p* < 0.05). Homozygous β0 genotypes correlated with lower cardiac T2^*^ (*p* = 0.009). Cardiac T2^*^ predicted β0 genotype with AUC of 0.812 (95% CI: 0.613–0.993), sensitivity 85.7%, and specificity 81.8% at a cutoff of 37.45 ms.

**Conclusion:**

EMH masses were highly prevalent in this TDT cohort. Larger EMH mass size—more so than greater number—was significantly associated with elevated cardiac T2^*^ values. Furthermore, cardiac T2^*^ serves as an effective non-invasive predictor of homozygous β0 genotypes in β-TDT patients.

## 1 Introduction

Globally, thalassemia ranks among the most prevalent monogenic disorders (1–3), with an age-standardized prevalence of 18.28 per 100,000 individuals reported in 2021 (3). Depending on the severity of anemia and the necessity for blood transfusions, thalassemia is categorized into transfusion-dependent thalassemia (TDT) and non-transfusion-dependent thalassemia (NTDT) (1). Chronic anemia, a hallmark of thalassemia, is a risk factor for extramedullary hematopoiesis (EMH), while iron overload contributes to organ dysfunction, particularly in the heart and liver (1). Magnetic resonance imaging (MRI) is essential for assessing EMH and organ iron deposition, guiding treatment strategies that improve patient outcomes (1).

Chronic anemia induces ineffective erythropoiesis, leading to EMH in patients with thalassemia. This compensatory mechanism, which forms mass-like structures, arises when the bone marrow's capacity to produce red blood cells (RBCs) fails to satisfy physiological needs (4–7). Notably, EMH can develop in the spinal canal, exerting pressure on the spinal cord and risking severe outcomes such as paraplegia (8–10).

Frequent blood transfusions, essential for managing thalassemia, result in significant iron deposition in critical organs, including the liver, heart, and endocrine glands (11). These transfusions are a fundamental cause of iron overload in TDT patients (12, 13). Furthermore, variations in the β-thalassemia genotype influence transfusion requirements and the spectrum of clinical complications. Particularly, the homozygous β0 genotype is associated with a range of serious health issues, such as heart failure and hypogonadotropic hypogonadism (14–16), underscoring the importance of genotype determination in managing the disease.

MRI is the primary modality for detecting, assessing, and tracking EMH (7). The MRI-T2^*^ technique stands as the benchmark for non-invasively quantifying tissue iron content (17, 18). Within this framework, cardiac T2^*^ values are essential for evaluating the extent of cardiac iron accumulation and the associated risk of cardiomyopathy in thalassemia major (TM) patients (19). A cardiac T2^*^ value < 10 ms substantially heightens the risk of heart failure, indicated by a relative risk (RR) of 160 (20). Innovations in iron chelation therapy have significantly advanced the treatment of iron buildup in these individuals (21, 22). A 10-year retrospective study involving 912 β-TDT patients showed that those with cardiac T2^*^ values < 27 ms faced an 8.6-fold increase in all-cause mortality risk (23). Another study noted a strong association between cardiac T2^*^ values < 34 ms and elevated all-cause mortality rates in β-thalassemia patients (24).

While previous studies have confirmed an inverse correlation between EMH presence and cardiac iron deposition (25, 26), no studies have quantified the impact of EMH burden (mass number/size) on cardiac iron content. Furthermore, identifying patients with high-risk homozygous β0 genotypes remains challenging in resource-limited regions, primarily due to structural inequities in regional and population-level genetic testing resources and technologies (27).

Given that EMH may have the capacity to sequester excess iron (25), and considering that cardiac T2^*^ values are lower in patients with homozygous β0 genotypes compared to those with β0β+ or homozygous β+ genotypes (28, 29), we hypothesized that EMH lesion size and number are associated with cardiac iron deposition, and that cardiac iron content could serve as a predictor of genotype in TDT patients. Additionally, we propose that EMH assessment could help guide clinical management decisions, specifically, prioritizing either the management of EMH-related mechanical compression effects on adjacent organs or enhancing cardiac iron monitoring and chelation therapy. Furthermore, cardiac iron content evaluation might provide clinical value in genotype prediction.

This study seeks to clarify the relationship between cardiac iron content and EMH masses in TDT patients, as well as to determine the potential of cardiac iron levels, as measured by the MRI-T2^*^ method, to predict homozygous β0 genotypes.

## 2 Materials and methods

### 2.1 Patients

Between October 2022 and June 2024, 77 patients with thalassemia were recruited for a prospective study. The inclusion criteria were as follows: (1) TDT patients were those who required regular blood transfusions. The remaining patients were classified as NTDT; (2) patients were aged 18 years or older; (3) patients provided written informed consent. Exclusion criteria included: (1) patients with contraindications to MRI; (2) patients unable to perform the breath-holding required during MRI scans; (3) pregnant patients; (4) patients diagnosed with malignant tumors; (5) patients who had undergone hematopoietic stem cell transplantation or gene therapy before enrollment; (6) patients whose image quality did not meet the requirements for quantification and diagnosis. The study adhered to the Declaration of Helsinki (revised 2013) and was approved by the institutional ethics committee (original clinical trial approval numbers: 2022-Y055-01 and 2022-Y034-01; supplemental approval number for data analysis: 2025-E0097).

### 2.2 MRI protocol

MRI was conducted using a Siemens 1.5 T MAGNETOM Altea Fit scanner equipped with a 12-channel abdominal coil. Cardiac MRI was performed with ECG gating, capturing a single midventricular septal short-axis slice during end-expiratory breath-hold, with a flip angle of 20°, a matrix of 67 mm × 224 mm, a field of view (FOV) of 239 mm × 400 mm, a slice thickness of 10 mm, a repetition time (TR) of 138 ms, and echo time (TE) of 2.97 ms, 5.54 ms, 8.23 ms, 10.92 ms, 13.61 ms, 16.30 ms, 18.99 ms, 21.68 ms, with a scan duration of around 10 s. Liver MRI was conducted at the level of the second porta hepatis during end-expiratory breath-hold, with a flip angle of 20°, a matrix of 64 mm × 128 mm, a FOV of 200 mm × 400 mm, a slice thickness of 10 mm, a TR of 200 ms, and TE of 1.33 ms, 2.40 ms, 3.48 ms, 4.63 ms, 5.71 ms, 6.86 ms, 7.94 ms, 9.09 ms, 10.17 ms, 11.32 ms, 12.40 ms, 13.55 ms, with a scan duration of about 14 s.

To detect EMH, scans included the chest, abdomen, pelvis, and spine. Sequences for the chest, abdomen, and pelvis included transverse T2-weighted FBLADE with fat saturation, transverse and coronal T2-weighted HASTE, and transverse T1-weighted VIBE Dixon (with in-phase, opposed-phase, water-only, and fat-only images); for the spine, included transverse and sagittal T2-weighted TSE, sagittal T2-weighted TSE with fat saturation, and sagittal T1-weighted TSE.

### 2.3 MR images post-processing and analysis

#### 2.3.1 Measurement of cardiac and liver T2^*^ values

Cardiac and liver T2^*^ values were measured using the thalassemia tools function in CMRtools software (version 2010). For cardiac measurements, a single region of interest (ROI) was positioned within the mid-ventricular septum, carefully avoiding the endocardium. For liver assessments, four ROIs were drawn at the level of the second porta hepatis, avoiding large vessels and bile ducts. After ROI delineation, T2^*^ values were calculated from the acquired signal intensity (SI) curves. A mono-exponential model was applied to quantify T2^*^ values. The fitting range was determined as follows: when all SI points across echo times (TEs) conformed to a mono-exponential decay curve, all echoes were used. However, if SI points at later TEs exhibited a “signal plateau” or deviated significantly from the curve, the truncation method was applied (30, 31): SI points were sequentially discarded backward until the remaining data points aligned with the mono-exponential decay model. Final T2^*^ values were accepted only if the goodness-of-fit (*R*^2^) reached ≥0.95. To assess inter-observer variability, three experienced radiologists (with over 3 years of experience in iron quantitative MRI) measured cardiac and liver T2^*^ values while being blinded to the corresponding patients' EMH status, genotypes, and each other's results. A cardiac T2^*^ value < 20 ms indicated cardiac iron overload, and a liver T2^*^ value < 6.3 ms suggested hepatic iron overload (18).

#### 2.3.2 Evaluation of EMH

Two other experienced radiologists (each with > 7 years of MR imaging diagnostic experience) independently evaluated EMH lesions, blinded to cardiac and liver T2^*^ values and patient genotypes. Evaluation content included: (1) anatomical location of EMH masses; (2) size of EMH masses, measured as the largest dimension (D_EMH_) among longitudinal diameter, transverse diameter, and height. Measurements were taken on axial T2-weighted sequences for longitudinal and transverse diameters, and on coronal or sagittal T2-weighted sequences for height; (3) the number of discrete EMH lesions (N_EMH_) with a maximum diameter ≥0.5 cm. In cases of disagreement regarding N_EMH_ and anatomical site, consensus was achieved through discussion. For D_EMH_, the radiologists independently recorded measurements, remaining blinded to each other's results.

### 2.4 Collection of data

Data collected included: (1) clinical characteristics, such as age, sex, body mass index (BMI), transfusion history, splenectomy history, and chelation therapy; (2) laboratory parameters, including hemoglobin, ferritin, and thalassemia genotypes—the average values of hemoglobin and ferritin levels within 3 months following the MRI were considered; thalassemia genotypes were obtained from patient medical records, referring to the β-genotype classification criteria reported in previous literature (32); (3) MRI data, which encompassed liver T2^*^ value, cardiac T2^*^ value, and EMH masses.

### 2.5 Sample size estimation

Sample size estimation was performed using an a priori power analysis (G^*^Power 3.1.9.7), based on an effect size (Cohen's d = 0.985) derived from previous literature (25). With α = 0.05 and statistical power = 0.80, a minimum sample size of 36 subjects was required.

### 2.6 Statistical analysis

Data analysis utilized SPSS 27. Intraclass correlation coefficient (ICC) was used to evaluate inter-observer reproducibility for cardiac T2^*^, liver T2^*^, and D_EMH_ measurements, with ICC > 0.75 indicating good agreement. The normality of continuous variables was assessed using the Shapiro-Wilk test and visual examination of histograms. Continuous variables that were normally distributed are presented as mean ± standard deviation (SD). Those with a non-normal distribution are presented as medians with interquartile ranges [median (P25, P75)]. Group comparisons involved the independent-sample *t*-test or the Mann-Whitney U test as appropriate. Categorical data were expressed as frequency (percentage) and analyzed via the Chi-square or Fisher's exact tests. To explore the independent effects of extramedullary hematopoiesis (EMH) characteristics on cardiac iron deposition, we constructed two multivariate linear regression models with cardiac T2^*^ value as the dependent variable. Model 1 was grouped by D_EMH_ and Model 2 by N_EMH_. Both models were adjusted for potential confounders, including age, gender, age at first transfusion, iron chelation duration, splenectomy status, duration post-splenectomy, pre-transfusion Hb level, and liver T2^*^ value. Regression results were presented as unstandardized coefficients (B) with 95% CIs, standardized coefficients (β), and *p*-values. Model fit was assessed by adjusted R^2^, and overall significance was tested by ANOVA. The capability of cardiac T2^*^ values to predict homozygous β0 genotypes in transfusion-dependent β-thalassemia patients was assessed using the receiver operating characteristic (ROC) curve. The area under the curve (AUC) was calculated to assess the predictive value of cardiac T2^*^ values for identifying homozygous β0 genotypes. Optimal cut-off values, sensitivity, and specificity were determined using the Youden index. Internal validation of the AUC was conducted using the bootstrap method with 5,000 iterations in R. A two-tailed *p*-value < 0.05 was considered statistically significant for all analyses.

## 3 Results

### 3.1 Patients' characteristics

A total of 77 thalassemia patients were included, consisting of 58 TDT and 19 NTDT patients. The median age was 22.5 years for TDT patients, 44.8% of whom were female, and 33 years for NTDT patients, with 57.9% being female.

Clinical and MR characteristics of the TDT and NTDT patients are detailed in Table 1. Notably, TDT patients were younger with significantly lower body mass index (*p* = 0.003 and *p* = 0.030, respectively). Higher proportions of TDT patients underwent chelation therapy and splenectomy (*p* < 0.001 and *p* = 0.006, respectively), compared to NTDT patients. Both groups were comparable in sex distribution and duration post-splenectomy (both *p* > 0.05).

**Table 1:** Comparison of clinical and MR characteristics between TDT patients and NTDT patients.

**Variable**	**TDT, *n* = 58**	**NTDT, *n* = 19**	***P*-value**
Female, *n* (%)	26 (44.8)	11 (57.9)	0.322
Age, years	22.5 (20, 31)	33 (23, 45)	**0.003**
BMI, kg/m^2^	18.81 ± 2.58	20.28 ± 2.33	**0.030**
Chelation received	52 (89.7)	4 (21.1)	<**0.001**
Splenectomy, *n* (%)	30 (51.7)	3 (15.8)	**0.006**
Duration post-splenectomy, years	13.97 ± 7.79	10.33 ± 7.10	0.444
Hb, g/L	83 (75, 91.25)	85.42 ± 11.14	0.232
0 < Hb < 95, *n* (%)	48 (82.8)	17 (89.5)	0.737
Hb ≥ 95, *n* (%)	10 (17.2)	2 (10.5)	
SF, μg/L	2336.00 (1081.33, 3885.88)	361.78 (278.67, 745.78)	<**0.001**
0 < SF < 1,000, *n* (%)	12 (20.7)	16 (84.2)	<**0.001**
SF ≥ 1,000, *n* (%)	46 (79.3)	3 (15.8)	
EMH masses, *n* (%)	42 (72.4)	6 (31.6)	**0.001**
Cardiac T2^*^, ms	35.81 (20.34, 46.92)	46.87 (39.89, 56.49)	**0.002**
Cardiac T2^*^ < 20 ms, *n* (%)	14 (24.1)	0(0)	**0.016**
Cardiac T2^*^>20 ms, *n* (%)	44 (75.9)	19 (100)	
Liver T2^*^, ms	1.27 (1.10, 3.07)	4.11 (2.12, 10.05)	<**0.001**
Liver T2^*^ < 6.3 ms, *n* (%)	52 (89.7)	11 (57.9)	**0.006**
Liver T2^*^>6.3 ms, *n* (%)	6 (10.3)	8 (42.1)	

Continuous variables are presented as mean ± SD or [median (P25, P75)]. Categorical data are expressed as *n* (%). Statistically significant results (*p* < 0.05) are highlighted in bold.

BMI, body mass index; Hb, hemoglobin; SF, serum ferritin; EMH, extramedullary hematopoiesis.

In laboratory parameters, TDT patients had significantly elevated serum ferritin (SF) levels (*p* < 0.001), with a higher proportion exceeding 1,000 μg/L (*p* < 0.001). Hemoglobin (Hb) levels and the proportion of patients with Hb ≥ 95 g/L showed no significant differences between groups.

MR results revealed a higher prevalence of EMH masses in TDT patients (*p* = 0.001), who also exhibited lower cardiac T2^*^ values (*p* = 0.002) and a greater proportion with cardiac T2^*^ values < 20 ms (*p* = 0.016). Liver T2^*^ values were lower in the TDT group (*p* < 0.001), with a higher incidence of liver T2^*^ values < 6.3 ms (*p* = 0.006).

### 3.2 Clinical and MR characteristics of TDT patients

**Figure 1:**
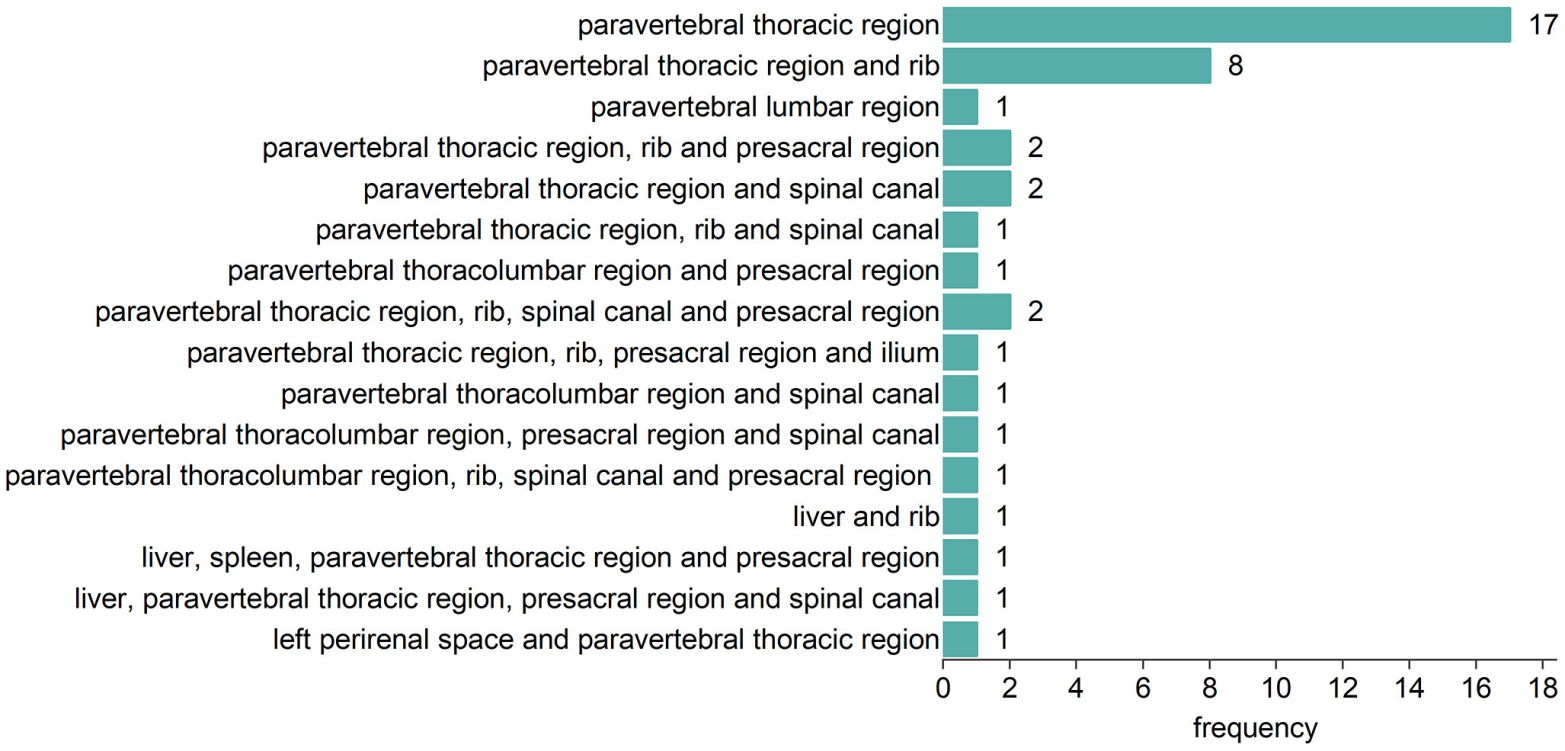
Anatomic distribution of EMH masses in 42 EMH (+) TDT patients.

**Figure 2:**
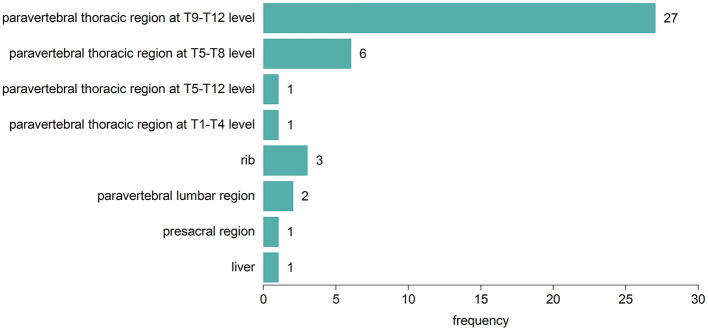
Anatomic sites of the largest EMH mass in each of the 42 EMH (+) TDT patients.

Fifty-eight TDT patients included 42 (72.4%) with EMH masses and 16 (27.5%) without. The anatomical distribution of EMH masses is depicted in Figure 1. The most common site was the paravertebral thoracic region, where EMH masses were identified in 40 (95.2%) of the TDT patients with EMH. EMH masses were also found within the spinal canal in 9 (21.4%) of these patients. Figure 2 illustrates the anatomical sites of the largest EMH mass for each patient with EMH. The largest masses were predominantly located in the lower paravertebral thoracic region at the T9-T12 level, involving 27 (64.3%) of the TDT patients with EMH.

Fifty-eight TDT patients were categorized into two groups: the EMH (+) group (*n* = 42) and the EMH (–) group (*n* = 16). For the EMH (+) group, the median maximum dimension of the largest EMH lesion measured per patient was 2.85 cm, with an interquartile range of 1.85–3.90 cm.

The EMH (+) TDT patients were categorized into two groups based on the N_EMH_: the 0 < N_EMH_ < 10 group (*n* = 13) and the N_EMH_ ≥ 10 group (*n* = 29). In addition, they were classified according to the median maximum dimension of EMH masses into the 0 < D_EMH_ < 2.85 cm group (*n* = 21) and the D_EMH_ ≥ 2.85 cm group (*n* = 21). Due to the small sample size (*n* = 19), NTDT patients were not categorized by EMH status.

#### 3.2.1 Association between EMH masses and cardiac T2^*^ values

Table 2 compares the clinical and MR characteristics between EMH (+) and EMH (-) TDT patients. Most characteristics were similar between the EMH (+) group and the EMH (–) group, except the EMH (+) group began their first transfusion later (*p* = 0.026) and had significantly higher cardiac T2^*^ values (*p* = 0.017).

**Table 2:** Comparison of clinical and MR characteristics between EMH (+) and EMH (–) TDT patients.

**Variable**	**EMH (+), *n* = 42**	**EMH (–), *n* = 16**	***P*-value**
Female, *n* (%)	16 (38.1%)	10 (62.5%)	0.095
Age, years	24 (20, 32.25)	22 (19, 25.75)	0.307
BMI, kg/m^2^	19.00 ± 2.82	18.36 ± 1.82	0.415
Age at first transfusion, months	60 (24, 126)	18 (8.5, 36)	**0.026**
Chelation received	38 (90.5)	14 (87.5)	1.000
Chelation duration	5.25 (1.71, 14.13)	11 (7.25, 17.50)	0.163
Splenectomy, *n* (%)	21 (50.0)	9 (56.3)	0.670
Duration post-splenectomy, years	14.14 ± 8.81	13.56 ± 5.10	0.854
Pre-transfusion Hb, g/L	83.50 (75.75, 90.25)	80.13 ± 20.73	0.931
0 < Hb < 95, *n* (%)	37 (88.1)	11 (68.8)	0.176
Hb ≥ 95, *n* (%)	5 (11.9)	5 (31.2)	
SF, μg/L	1,610 (1005.50, 3455.25)	3869.82 ± 2756.70	0.068
0 < SF < 1,000, *n* (%)	10 (23.8)	2 (12.5)	0.557
SF ≥ 1,000, *n* (%)	32 (76.2)	14 (87.5)	
Cardiac T2^*^, ms	37.22 ± 16.08	25.54 ± 16.36	**0.017**
Cardiac T2^*^ < 20 ms, *n* (%)	7 (16.7)	7 (43.8)	0.070
Cardiac T2^*^>20 ms, *n* (%)	35 (83.3)	9 (56.2)	
Liver T2^*^, ms	1.40 (1.09, 3.01)	1.26 (1.10, 3.78)	0.821
Liver T2^*^ < 6.3 ms, *n* (%)	37 (88.1)	15 (93.7)	0.881
Liver T2^*^>6.3 ms, *n* (%)	5 (11.9)	1 (6.3)	

Continuous variables are presented as mean ± SD or [median (P25, P75)]. Categorical data are expressed as *n* (%). Statistically significant results (*p* < 0.05) are highlighted in bold.

BMI, body mass index; Hb, hemoglobin; SF, serum ferritin; EMH, extramedullary hematopoiesis.

#### 3.2.2 Correlation of the number and size of EMH masses with cardiac T2^*^ values

Subgroup analyses were conducted for EMH (+) TDT patients (Table 3). The cardiac T2^*^ values were significantly lower in the 0 < N_EMH_ < 10 group compared to the N_EMH_ ≥ 10 group (*p* = 0.018). No notable differences were found between groups for the proportion of patients with a cardiac T2^*^ value < 20 ms, liver T2^*^ values, or age at the first transfusion (all *p* > 0.05). The 0 < D_EMH_ < 2.85 cm group displayed lower cardiac T2^*^ values compared to the D_EMH_ ≥ 2.85 cm group (*p* = 0.014). No significant differences were found between these two groups regarding the proportions of patients with a cardiac T2^*^ value < 20 ms, liver T2^*^ values, or the proportions of patients with a liver T2^*^ value < 6.3 ms (all *p* > 0.05). Furthermore, the age at the first transfusion did not differ significantly between the groups.

**Table 3:** Comparison of clinical and MR characteristics of TDT patients with EMH masses between subgroups based on the EMH masses in number and size.

**Variable**	**0 < N_EMH_ < 10, *n* = 13**	**N_EMH_ ≥10, *n* = 29**	***P*-value**	**0 < D_EMH_ < 2.85, *n* = 21**	**D_EMH_ ≥2.85, *n* = 21**	***P*-value**
Age at first transfusion, months	48 (18, 114)	72 (24, 144)	0.567	36 (21, 108)	96 (30, 144)	0.194
Cardiac T2^*^, ms	28.57 ± 16.61	41.09 ± 14.49	**0.018**	31.21 ± 16.52	43.22 ± 13.46	**0.014**
Cardiac T2^*^ < 20 ms, *n* (%)	4 (30.8)	3 (10.3)	0.232	5 (23.8)	2 (9.5)	0.408
Cardiac T2^*^>20 ms, *n* (%)	9 (69.2)	26 (89.7)		16 (76.2)	19 (90.5)	
Liver T2^*^, ms	1.12 (1.05, 3.37)	1.77 (1.13, 3.15)	0.314	1.77 (1.09, 6.34)	1.25 (1.08, 2.60)	0.399
Liver T2^*^ < 6.3 ms, *n* (%)	11 (84.6)	26 (89.7)	1.000	16 (76.2)	21 (100)	0.057
Liver T2^*^>6.3 ms, *n* (%)	2 (15.4)	3 (10.3)		5 (23.8)	0 (0)	

Continuous variables are presented as mean ± SD or [median (P25, P75)]. Categorical data are expressed as *n* (%). Statistically significant results (*p* < 0.05) are highlighted in bold.

To further assess the independent association of EMH characteristics with cardiac T2^*^, we constructed two multivariable linear regression models (Table 4). Model 1 (grouped by D_EMH_) was statistically significant overall (*F* = 2.833, *p* = 0.008), with an adjusted *R*^2^ of 0.243, indicating that the model explains 24.3% of the variation in cardiac T2^*^ values. After adjusting for all covariates, the presence of D_EMH_ ≥ 2.85 cm was independently associated with higher cardiac T2^*^ values (B = 13.651, 95% CI: 2.290 to 25.012, *p* = 0.020) compared to the no EMH group. Model 2 (grouped by N_EMH_) was also statistically significant overall (*F* = 2.514, *p* = 0.016), with an adjusted *R*^2^ of 0.210, indicating that the set of independent variables collectively explains 21% of the variation in cardiac T2^*^ values. However, after adjusting for other variables, no independent variable was identified as a significant predictor (all *p* > 0.05).

**Table 4:** Multivariable linear regression analysis of factors associated with cardiac T2^*^.

**Variable**	**Model 1**			**Model 2**		
	**B (95% CI)**	β	***P***	**B (95% CI)**	β	***P***
**EMH group**						
D_EMH_ < 2.85 cm group	0.814 (−9.946, 11.573)	0.023	0.880	–	–	–
D_EMH_ ≥ 2.85 cm group	**13.651 (2.290, 25.012)**	**0.393**	**0.020**	–	–	–
N_EMH_ < 10 group	–	–	–	0.649 (−11.273, 12.572)	0.016	0.913
N_EMH_ ≥ 10 group	–	–	–	10.156 (−0.395, 20.707)	0.304	0.059
**Covariates**						
Age, years	0.316 (−0.331, 0.964)	0.166	0.331	0.481 (−0.154, 1.116)	0.252	0.134
Gender	7.692 (−1.628, 17.011)	0.229	0.103	8.825 (−0.609, 18.259)	0.263	0.066
Age at first transfusion, months	−0.020 (−0.069, 0.029)	−0.141	0.422	−0.018 (−0.068, 0.033)	−0.125	0.487
Iron chelation duration, years	−0.360 (−0.983, 0.263)	−0.164	0.251	−0.250 (−0.908, 0.407)	−0.114	0.448
Splenectomy	−5.582 (−19.886, 8.721)	−0.167	0.436	−6.657 (−21.223, 7.909)	−0.199	0.363
Duration post-splenectomy, years	−0.175 (−1.041, 0.691)	−0.093	0.687	−0.042 (−0.906, 0.823)	−0.022	0.097
Pre-transfusion Hb, g/L	−0.083 (−0.378, 0.212)	−0.070	0.576	−0.053 (−0.352, 0.246)	−0.045	0.722
Liver T2^*^, ms	0.726 (−0.350, 1.801)	0.190	0.181	0.306 (−0.692, 1.304)	0.080	0.540
**Model fit statistics**						
Adjusted *R*^2^	0.243			0.210		
*F*	2.833			2.514		
Model *p*-value	**0.008**			**0.016**		

B, unstandardized coefficient; CI, confidence interval; β, standardized coefficient; EMH, extramedullary hematopoiesis; Hb, hemoglobin.

Statistically significant results (*p* < 0.05) are highlighted in bold. The EMH groups are compared to the reference group of patients with no EMH.

### 3.3 Characteristics of TDT patients with different β genotypes

Thalassemia genotypes were collected in 25 of the 58 TDT patients. CD41-41, CD17, and−28 were the most frequent mutations (Table 5). CD41-42/CD41-42, CD41-42/-28, and CD17/CD41-42 were the most prevalent genotypes. The homozygous β0 genotypes occurred more frequently (Table 6).

**Table 5:** Frequency of different mutations in transfused-dependent β-thalassemia patients.

**Mutation**	**Frequency**
CD41-42	28
CD17	8
−28	7
IVS-I-1	1
CD43	1
βE	3
−90	1
CD71-72	1

**Table 6:** Frequency of different genotypes in transfused-dependent β-thalassemia patients.

**Genotypes**	**Group**	**Frequency**
CD41-42/CD41-42	β0β0	7
CD17/CD41-42	β0β0	3
CD41-42/IVS-I-1	β0β0	1
CD17/CD17	β0β0	1
CD41-42/CD43	β0β0	1
−28/CD17	β0β+	2
CD17/βE	β0β+	1
CD41-42/βE	β0β+	2
CD41-42/-28	β0β+	5
CD41-42/-90	β0β+	1
CD71-72/CD41-42	β0β0	1

Patients were categorized into the homozygous β0 group (*n* = 14) and the heterozygous β0β+ group (*n* = 11) based on each allele's phenotypic expression (β0 or β+). A comparison of clinical and MR characteristics was conducted between the two groups.

#### 3.3.1 Analyses of clinical and MR characteristics between β-thalassemia patients with homozygous β0 and heterozygous β0β+ genotypes

**Figure 3:**
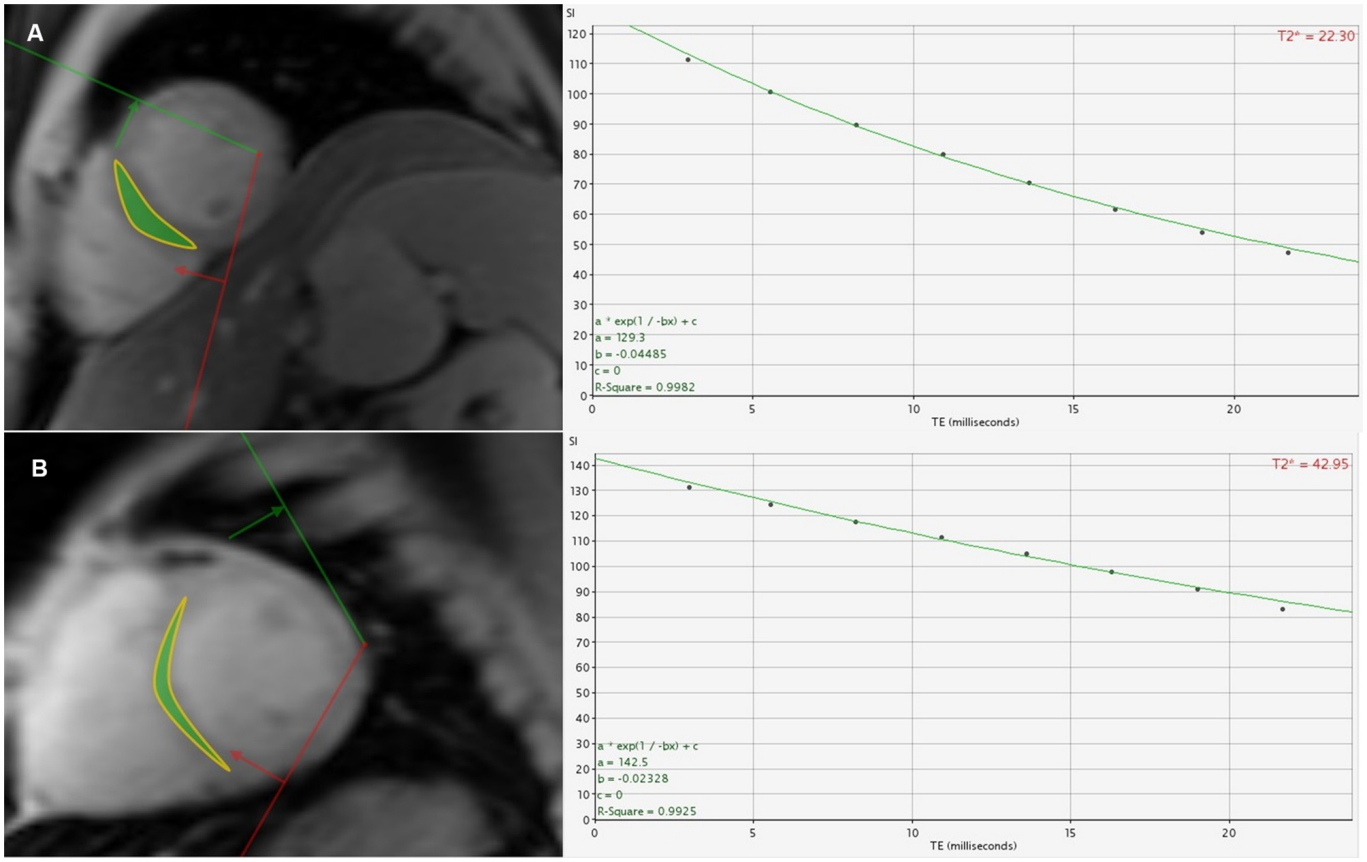
**(A)** A 26-year-old male β-TDT patient with the homozygous β0 genotypes had a cardiac T2* value of 22.30 ms. **(B)** A 31-year-old male β-TDT patient with the heterozygous β0β+ genotypes had a cardiac T2* value of 42.95 ms.

Table 7 outlines the differences between patients with homozygous β0 and heterozygous β0β+ genotypes. The homozygous β0 group displayed significantly lower cardiac T2^*^ values (*p* = 0.009) (Figure 3). Additionally, this group had a higher proportion of patients with a cardiac T2^*^ value < 20 ms (*p* = 0.033). In contrast, liver T2^*^ values and the proportion of patients with a liver T2^*^ value < 6.3 ms did not significantly differ between the two groups (both *p* > 0.05). There were no significant differences between the two groups in terms of gender, age, BMI, history of splenectomy, duration post-splenectomy, chelation therapy, chelation duration, pre-transfusion Hb, and SF (all *p* > 0.05).

**Table 7:** Comparison of clinical and MR characteristics between patients with β0β0 genotypes and β0β+ genotypes.

**Variable**	**β0β0, *n* = 14**	**β0β+, *n* = 11**	***P*-value**
Female, *n* (%)	6 (42.9)	4 (36.4)	1.000
Age, years	22.57 ± 3.65	27.91 ± 8.34	0.069
BMI, kg/m^2^	19.83 ± 3.11	18.16 ± 2.13	0.143
Age at first transfusion, months	24 (12, 57)	36 (18, 48)	0.472
Chelation received	14 (100)	11(100)	1
Chelation Duration	13.5 (8.75, 17.25)	7 (2, 17)	0.239
Splenectomy, *n* (%)	12 (85.7)	6 (54.5)	0.177
Duration post-splenectomy, years	11.33 ± 5.00	18.67 ± 10.19	0.270
Pre-transfusion Hb, g/L	87.43 ± 10.83	77.91 ± 17.90	0.113
0 < Hb < 95, *n* (%)	10 (71.4)	10 (90.9)	0.341
Hb ≥ 95, *n* (%)	4 (28.6)	1(9.1)	
SF, μg/L	3291.66 ± 2272.53	1940.50 (1280.90, 3763.00)	0.547
0 < SF < 1,000, *n* (%)	2 (14.3)	2 (18.2)	1.000
SF ≥ 1,000, *n* (%)	12 (85.7)	9 (81.8)	
EMH masses, *n* (%)	8 (57.1)	8 (72.7)	0.677
Cardiac T2^*^, ms	20.25 ± 16.06	44.08 (39.58, 46.45)	**0.009**
Cardiac T2^*^ < 20 ms, *n* (%)	8 (57.1)	1 (9.1)	**0.033**
Cardiac T2^*^>20 ms, *n* (%)	6 (42.9)	10 (90.9)	
Liver T2^*^, ms	1.21 (1.11, 3.33)	1.21 (1.11, 2.87)	0.763
Liver T2^*^ < 6.3 ms, *n* (%)	13 (92.9)	10 (90.9)	1.000
Liver T2^*^>6.3 ms, *n* (%)	1 (7.1)	1 (9.1)	

Continuous variables are presented as mean ± SD or [median (P25, P75)]. Categorical data are expressed as *n* (%). Statistically significant results (*p* < 0.05) are highlighted in bold.

BMI, body mass index; Hb, hemoglobin; SF, serum ferritin; EMH, extramedullary hematopoiesis.

#### 3.3.2 Cardiac T2^*^ values for predicting the homozygous β0 genotypes

**Figure 4:**
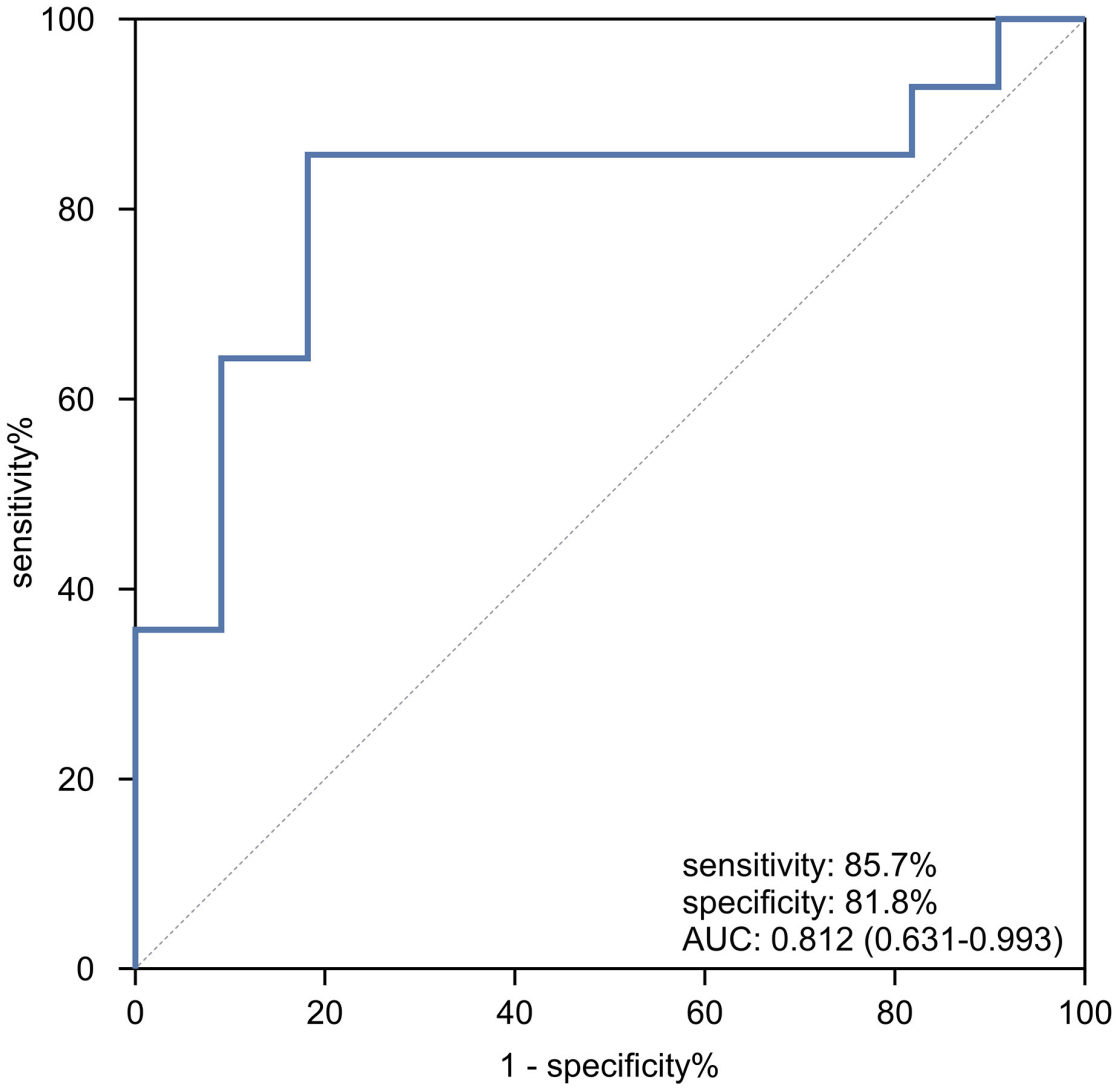
The ROC curve analysis of cardiac T2* for predicting patients with homozygous β0 genotypes in TD β-thalassemia patients.

In the genotyped subset of patients (*n* = 25), a ROC curve was generated to assess the ability of cardiac T2^*^ to differentiate between homozygous β0 and heterozygous β0β+ genotypes. As shown in Figure 4, the AUC was 0.812 (95% CI: 0.613–0.993, *p* = 0.009). The optimal cardiac T2^*^ cutoff value for predicting homozygous β0 genotypes was identified as 37.45 ms, with sensitivity, specificity, accuracy, positive predictive value, and negative predictive value of 85.7%, 81.8%, 84.0%, 85.7%, and 81.8%, respectively. Internal validation of the model was performed using bootstrap resampling with 5,000 repetitions. The results showed a calibrated AUC of 0.812 (95% CI: 0.604–0.981).

### 3.4 ICC for inter-observer reproducibility for cardiac T2^*^, liver T2^*^, and D_*EMH*_

The inter-observer reproducibility for cardiac T2^*^, liver T2^*^, and D_EMH_ was excellent (ICC: 0.989-0.997, *p* < 0.001) (Table 8).

**Table 8:** ICC for inter-observer reproducibility for cardiac T2^*^, liver T2^*^, and D_EMH_.

**Parameter**	**ICC**	**95% confidence interval**	***P***
cardiac T2^*^	0.989	[0.984, 0.993]	< 0.001
liver T2^*^	0.992	[0.988, 0.995]	< 0.001
D_EMH_	0.997	[0.995, 0.998]	< 0.001

## 4 Discussion

This prospective study reveals a notably high prevalence of EMH masses among TDT patients, which correlates significantly with reduced cardiac iron deposition. Notably, the size of EMH masses demonstrated a stronger association with elevated cardiac T2^*^ than did the number of masses, suggesting that larger ectopic hematopoietic foci may play a more critical role in mitigating myocardial iron overload. Furthermore, cardiac T2^*^ showed strong predictive performance for identifying homozygous β0 genotypes in β-TDT patients. These findings highlight the clinical relevance of systematically assessing both EMH burden and cardiac iron levels in TDT patients. Cardiac T2^*^ may serve as a non-invasive indicator to support genotype prediction and facilitate personalized treatment strategies, particularly in settings where genetic testing is limited.

Previous studies have reported EMH prevalence rates ranging from 4.4% to 27% among TDT patients (25, 26, 33–35). However, in this study, a significantly higher prevalence of 72.4% was observed, which exceeds previous reports. This discrepancy may be attributed not only to different sample sizes between studies, but also to the suboptimal transfusion support in our cohort of TDT patients. The median pre-transfusion hemoglobin level in our TDT cohort was 83 g/L, substantially lower than the recommended target of ≥ 95 g/L required to effectively suppress erythropoiesis and prevent EMH (1, 13). This likely stems from broader systemic challenges such as economic constraints and limited blood supply (36). Consequently, our study cohort presents a unique natural experiment demonstrating that TDT patients with inadequate transfusion management can exhibit an EMH prevalence pattern that mirrors, or even exceeds, that seen in NTDT. This finding underscores the critical importance of adhering to recommended transfusion guidelines to mitigate the risk of EMH and its associated complications.

Our study further revealed that TDT patients had higher levels of cardiac and hepatic iron compared to NTDT patients, despite NTDT patients being older on average. This difference is likely attributable to the distinct mechanisms underlying iron overload in each group: repetitive RBC transfusions in TDT patients vs. increased intestinal iron absorption in NTDT patients (12, 13). TDT patients accumulate 6–10 g of iron annually through transfusions, whereas NTDT patients accumulate only 2–5 g through intestinal absorption (37). This greater iron burden in TDT patients underscores the necessity for more aggressive iron chelation therapy compared to NTDT patients. We observed higher SF levels in TDT patients compared to NTDT patients, primarily due to the significantly higher exogenous iron intake required for SF synthesis in TDT patients. Pathophysiologically, ferritin is predominantly stored in the reticuloendothelial system and is released into the serum as needed. In NTDT patients, ineffective erythropoiesis and chronic anemia mediate a signaling pathway that reduces hepcidin levels, thereby promoting iron release from the reticuloendothelial system, which leads to decreased ferritin synthesis and release. In contrast, in TDT patients, iron is preferentially distributed to the reticuloendothelial system, enhancing ferritin synthesis and increasing serum ferritin levels (38, 39).

EMH, typically manifesting as masses, is formed by hematopoietic tissues outside the bone marrow to compensate for its inadequate function (7). The paraspinal region is most frequently involved (7, 40). A retrospective study of 1,266 TDT patients showed that 98.8% (165/167) of EMH (+) TM patients had EMH masses in the paravertebral thoracic region (26), corroborating our findings where 95.2% (40/42) of EMH (+) TDT patients were affected in this area. A lower prevalence was reported by Huang et al. (41), who observed EMH in the paraspinal region in 80% of 26 NTDT patients. In our cohort, the largest EMH masses were predominantly found in the lower paravertebral regions of the thoracic spine. Given that the size of EMH masses correlates with their duration of growth, it is plausible to suggest that EMH preferentially develops in this specific area of the thoracic spine. Given that the scanning range for cardiac and liver iron quantification includes parts of the lower thoracic spine, it is essential to incorporate the assessment of paravertebral EMH masses into these imaging reports for effective monitoring and follow-up. In our EMH (+) TDT cohort, the presence of EMH masses within the spinal canal was notable, with a prevalence of 21.4%. Fortunately, no clinical signs of spinal cord compression were observed during this study.

The relationship between EMH masses and cardiac iron content in TDT patients remains controversial (5). Ricchi et al. (25, 26) observed higher cardiac T2^*^ values in TDT patients with EMH masses than those without EMH masses (*p* < 0.01). However, Sousos et al. (33) reported no significant difference in cardiac T2^*^ values between EMH (+) and EMH (–) TDT patients. Our study adds nuance to this discussion by demonstrating that the association is specifically driven by the size rather than the mere presence or number of EMH masses. After adjustment for confounders, the presence of larger EMH masses was independently associated with significantly higher cardiac T2^*^ values (B = 13.95, *p* = 0.023), whereas a greater number of masses showed only a non-significant trend (*p* = 0.056). This suggests that the larger masses may be more effective at sequestering iron than multiple smaller ones, possibly due to greater iron avidity in larger hematopoietic tissues (25), thereby reducing its deposition in the heart. To our knowledge, this is the first study to quantitatively demonstrate that EMH size is a more robust predictor of cardiac iron offloading than EMH number. These insights highlight the importance of characterizing EMH morphology in addition to its presence and underscore the need for vigilant monitoring of these masses to prevent severe complications due to compression by EMH masses.

Previous research has indicated that homozygous β0 genotypes are associated with a higher incidence of clinical complications compared to other genotypes (14–16). Understanding the β-genotype's association with clinical severity is crucial for developing personalized treatment plans. There are limited studies on the predictive value of cardiac T2^*^ for homozygous β0 genotypes in TD β-thalassemia patients, according to our knowledge. In our study, CD41-41, CD17, and −28 were the most common mutations, aligning with findings (42), who reviewed the global distribution of β-thalassemia mutations. In our study, TDT patients with the homozygous β0 genotypes demonstrated significantly lower cardiac T2^*^ values compared to those with the heterozygous β0β+ genotypes (*p* = 0.009), and a higher proportion of these patients had cardiac T2^*^ values < 20 ms.

These findings are consistent with those from the other study (28), which reported significantly lower median cardiac T2^*^ values in patients with homozygous β0 genotypes compared to β0β+ genotypes (*p* < 0.05). Another study (29) observed a higher proportion of patients with cardiac T2^*^ values < 20 ms in the β0β0 group, although the *p*-value was not specified. Similarly, Zhou et al. (43) found a significantly higher incidence of cardiac T2^*^ values < 20 ms in β0β0 patients compared to β0β+ patients (*p* < 0.001). In our study, analysis using the ROC curve and Jordan index confirmed that the optimal cutoff value of 37.45 ms yielded an AUC of 0.812 (95% CI: 0.613–0.993), with sensitivity, specificity, and accuracy of 85.7%, 81.8%, and 84.0%, respectively. This suggests that cardiac T2^*^ values could be useful for predicting homozygous β0 genotypes in β-TDT patients. Homozygous β0 genotypes are associated with several complications, including heart failure (14–16). However, many thalassemia patients at their initial visit for cardiac and hepatic iron content monitoring cannot provide a definitive genetic test report due to economic constraints or a lack of understanding about thalassemia genotypes. The ability of cardiac iron content, assessed via MR-T2^*^ technique, to predict homozygous β0 genotypes could offer valuable insights for clinical decision-making.

This study presents several innovations. First, it prospectively quantifies, for the first time, the association between EMH burden (number/size of masses) and cardiac iron deposition, addressing a significant gap in previous research. Second, it uniquely demonstrates the predictive value of cardiac T2^*^ for identifying homozygous β° genotypes in TDT patients, providing a valuable alternative for resource-limited regions lacking genetic testing capabilities. Finally, the blinded evaluation approach ensures the reliability of quantitative data. However, several limitations exist. First, potential myocardial inflammation or fibrosis, pathologies that may coexist with EMH and cause myocardial remodeling, were not accounted for, which might confound cardiac T2^*^ values. Second, due to missing data regarding total transfusion volumes and detailed chelation therapy histories, differences in organ iron deposition between subgroups must be interpreted cautiously. Third, the utility of cardiac T2^*^ for predicting homozygous β0 genotypes remains limited to populations with established β-thalassemia diagnoses. Moreover, T2^*^ measurements are technique-sensitive and influenced by MRI parameters and magnetic field strength, implying that optimal genotypic thresholds may vary across scanners. Crucially, although the predictive model demonstrated encouraging discrimination (AUC = 0.812), it was derived from a small sample (*n* = 25). These limitations affect the precision and generalizability of the proposed cutoff value (37.45 ms). Therefore, our findings should be interpreted as preliminary and exploratory, suggesting a plausible association rather than providing a definitive diagnostic tool. Longitudinal, multicenter studies with larger cohorts are needed to validate these findings.

## 5 Conclusion

In this cohort of TDT patients, EMH masses were highly prevalent, largely attributable to suboptimal transfusion support leading to inadequate suppression of erythropoiesis. Importantly, larger—rather than more numerous—EMH masses were more strongly associated with reduced cardiac iron deposition, suggesting that mass size may be a more critical factor in mitigating myocardial iron overload. These findings may provide insight into the systematic surveillance of EMH masses to optimize iron chelation strategies and to prevent compression-related complications associated with large EMH size and in TDT patients. Furthermore, a cardiac T2^*^ cutoff of 37.45 ms shows promise as a non-invasive predictor of homozygous β° genotypes in β-TDT patients, offering potential utility in resource-limited settings where genetic testing is unavailable. However, this threshold requires further validation in larger, multicenter studies to confirm its diagnostic accuracy and generalizability.

## Data Availability

The original contributions presented in the study are included in the article/supplementary material, further inquiries can be directed to the corresponding author.

## References

[1] Kattamis A Kwiatkowski JL Aydinok Y. Thalassaemia. Lancet. (2022) 399:2310–24. doi: 10.1016/S0140-6736(22)00536-0 35691301

[2] Piel FB Weatherall DJ. The α-thalassemias. N Engl J Med. (2014) 371:1908–16. doi: 10.1056/NEJMra1404415 25390741

[3] Tuo Y Li Y Li Y Ma J Yang X Wu S . Global, regional, and national burden of thalassemia, 1990-2021: a systematic analysis for the global burden of disease study 2021. EClinicalMedicine. (2024) 72:102619. doi: 10.1016/j.eclinm.2024.102619 38745964 PMC11090906

[4] Subahi EA Ata F Choudry H Iqbal P AlHiyari MA Soliman AT . Extramedullary haematopoiesis in patients with transfusion dependent β-thalassaemia (TDT): a systematic review. Ann Med. (2022) 54:764–74. doi: 10.1080/07853890.2022.2048065 35261317 PMC8941948

[5] Yang X Chen D Long H Zhu B. The mechanisms of pathological extramedullary hematopoiesis in diseases. Cell Mol Life Sci. (2020) 77:2723–38. doi: 10.1007/s00018-020-03450-w 31974657 PMC11104806

[6] Yamamoto K Miwa Y Abe-Suzuki S Abe S Kirimura S Onishi I . Extramedullary hematopoiesis: elucidating the function of the hematopoietic stem cell niche (Review). Mol Med Rep. (2016) 13:587–91. doi: 10.3892/mmr.2015.4621 26648325

[7] Haidar R Mhaidli H Taher AT. Paraspinal extramedullary hematopoiesis in patients with thalassemia intermedia. Eur Spine J. (2010) 19:871–8. doi: 10.1007/s00586-010-1357-2 20204423 PMC2899982

[8] de la Iglesia Iñigo S Navarrete Bullón L Stuckey R Veiga Vaz Á Perera MDM Hernández Hernández M . Cauda equina syndrome secondary to extramedullary erythropoiesis in a transfusion-dependent thalassemia patient following treatment with luspatercept: a case report. Br J Haematol. (2022) 199:e30–3. doi: 10.1111/bjh.18470 36161438

[9] Dragean CA Duquesne L Theate I Ghaye B Coche EE. Extramedullary haemopoiesis and spinal cord compression. Lancet. (2011) 377:251. doi: 10.1016/S0140-6736(10)60485-0 21111477

[10] Malik M Pillai LS Gogia N Puri T Mahapatra M Sharma DN . Paraplegia due to extramedullary hematopoiesis in thalassemia treated successfully with radiation therapy. Haematologica. (2007) 92:28–30. doi: 10.3324/haematol.10199 17405752

[11] Taher AT Musallam KM Cappellini MD. β-Thalassemias. N Engl J Med. (2021) 384:727–43. doi: 10.1056/NEJMra2021838 33626255

[12] Porter JB Garbowski M. The pathophysiology of transfusional iron overload. Hematol Oncol Clin North Am. (2014) 28:683–701. doi: 10.1016/j.hoc.2014.04.003 25064708

[13] Cappellini MD Farmakis D Porter J Taher A. 2021 Guidelines: For the MANAGEMENT of Transfusion Dependent Thalassaemia (TDT). Nicosia: Thalassaemia International Federation (2023). 38683909

[14] Meloni A Pistoia L Ricchi P Bagnato S Longo F Messina G . Impact of genotype on multi-organ iron and complications in patients with non-transfusion-dependent β-thalassemia intermedia. Ann Hematol. (2024) 103:1887–96. doi: 10.1007/s00277-024-05741-9 38581547

[15] Al-Akhras A Badr M El-Safy U Kohne E Hassan T Abdelrahman H . Impact of genotype on endocrinal complications in β-thalassemia patients. Biomed Rep. (2016) 4:728–36. doi: 10.3892/br.2016.646 27284414 PMC4887852

[16] Skordis N Michaelidou M Savva SC Ioannou Y Rousounides A Kleanthous M . The impact of genotype on endocrine complications in thalassaemia major. Eur J Haematol. (2006) 77:150–6. doi: 10.1111/j.1600-0609.2006.00681.x 16800840

[17] Reeder SB Yokoo T França M Hernando D Alberich-Bayarri Á Alústiza JM . Quantification of liver iron overload with MRI: review and guidelines from the ESGAR and SAR. Radiology. (2023) 307:221856. doi: 10.1148/radiol.221856 36809220 PMC10068892

[18] Di Tucci AA Matta G Deplano S Gabbas A Depau C Derudas D . Myocardial iron overload assessment by T2^*^ magnetic resonance imaging in adult transfusion dependent patients with acquired anemias. Haematologica. (2008) 93:1385–8. doi: 10.3324/haematol.12759 18603557

[19] Vlachou M Kamperidis V Giannakoulas G Karamitsos T Vlachaki E Karvounis H. Biochemical and imaging markers in patients with thalassaemia. Hellenic J Cardiol. (2021) 62:4–12. doi: 10.1016/j.hjc.2020.04.012 32387594

[20] Kirk P Roughton M Porter JB Walker JM Tanner MA Patel J . Cardiac T2^*^ magnetic resonance for prediction of cardiac complications in thalassemia major. Circulation. (2009) 120:1961–8. doi: 10.1161/CIRCULATIONAHA.109.874487 19801505 PMC2784198

[21] Nichols-Vinueza DX White MT Powell AJ Banka P Neufeld EJ MRI. guided iron assessment and oral chelator use improve iron status in thalassemia major patients. Am J Hematol. (2014) 89:684–8. doi: 10.1002/ajh.23715 24652616 PMC5752110

[22] Pines M Kleinert D Thomas C Mensah C Musallam KM Sheth S. Real-world experience with iron chelation therapy in transfusion-dependent thalassemia: impact of the oral chelators' era. Ann Hematol. (2024) 103:5229–34. doi: 10.1007/s00277-024-06092-1 39672943

[23] Musallam KM Barella S Origa R Ferrero GB Lisi R Pasanisi A . Revisiting iron overload status and change thresholds as predictors of mortality in transfusion-dependent β-thalassemia: a 10-year cohort study. Ann Hematol. (2024) 103:2283–97. doi: 10.1007/s00277-024-05715-x 38503936

[24] Kamperidis V Vlachou M Pappa Z Pantelidou D Karamitsos T Papadopoulou D . Prediction of long-term survival in patients with transfusion-dependent hemoglobinopathies: insights from cardiac imaging and ferritin. Hellenic J Cardiol. (2021) 62:429–38. doi: 10.1016/j.hjc.2021.01.010 33524617

[25] Ricchi P Ammirabile M Spasiano A Costantini S Di Matola T Pepe A . Extramedullary haematopoiesis correlates with genotype and absence of cardiac iron overload in polytransfused adults with thalassaemia. Blood Transfus. (2014) 12:124–30. doi: 10.2450/2013.0287-12 24120603 PMC3934250

[26] Ricchi P Meloni A Spasiano A Neri MG Gamberini MR Cuccia L . Extramedullary hematopoiesis is associated with lower cardiac iron loading in chronically transfused thalassemia patients. Am J Hematol. (2015) 90:1008–12. doi: 10.1002/ajh.24139 26228763

[27] Wang WD Hu F Zhou DH Gale RP Lai YR Yao HX . Thalassaemia in China. Blood Rev. (2023) 60:101074. doi: 10.1016/j.blre.2023.101074 36963988

[28] Pistoia L Meloni A Salvadori S Spasiano A Lisi R Rosso R . Cardiac involvement by CMR in different genotypic groups of thalassemia major patients. Blood Cells Mol Dis. (2019) 77:1–7. doi: 10.1016/j.bcmd.2019.01.008 30878912

[29] Pistoia L Meloni A Ricchi P Filosa A Lisi R Maggio A . Genotypic groups as risk factors for cardiac magnetic resonance abnormalities and complications in thalassemia major: a large, multicentre study. Blood Transfus. (2021) 19:168–76. doi: 10.2450/2020.0023-20 33000750 PMC7925229

[30] He T Gatehouse PD Smith GC Mohiaddin RH Pennell DJ Firmin DN. Myocardial T2^*^ measurements in iron-overloaded thalassemia: an *in vivo* study to investigate optimal methods of quantification. Magn Reson Med. (2008) 60:1082–9. doi: 10.1002/mrm.21744 18956471 PMC2593631

[31] Luo C Peng F Xu F Tang C Zhang Y Huang C . Assessing the accuracy of CMRtools software for diagnosing liver iron overload in thalassemia patients: influencing factors and optimisation strategies. Front Med (Lausanne). (2024) 20:1424294. doi: 10.3389/fmed.2024.1424294 39371340 PMC11449772

[32] Shang X Peng Z Ye Y. Asan, Zhang X, Chen Y, et al. Rapid targeted next-generation sequencing platform for molecular screening and clinical genotyping in subjects with hemoglobinopathies. EBioMedicine. (2017) 23:150–9. doi: 10.1016/j.ebiom.2017.08.015 28865746 PMC5605365

[33] Sousos N Adamidou D Klonizakis P Agapidou A Theodoridou S Spanos G . Presence of the IVS-I-6-mutated allele in beta-thalassemia major patients correlates with extramedullary hematopoiesis incidence. Acta Haematol. (2017) 137:175–82. doi: 10.1159/000463919 28399542

[34] Ricchi P Meloni A Grigoratos C Toia P Fina P Pistoia L . Prevalence of extramedullary hematopoiesis, renal cysts, splenic and hepatic lesions, and vertebral hemangiomas among thalassemic patients: a retrospective study from the Myocardial Iron Overload in Thalassemia (MIOT) network. Ann Hematol. (2019) 98:1333–9. doi: 10.1007/s00277-019-03659-1 30891614

[35] Chapchap EC Silva MMA Baroni RH Araujo ADS de Assis RA Loggetto SR . Extramedullary haematopoiesis in patients with thalassemia: a cross-sectional description of its prevalence, clinical features and survival. Hematol Transfus Cell Ther. (2024) 46 Suppl 5:143–51. doi: 10.1016/j.htct.2023.07.005 37690980 PMC11670632

[36] Zhen X Ming J Zhang R Zhang S Xie J Liu B . Economic burden of adult patients with β-thalassaemia major in mainland China. Orphanet J Rare Dis. (2023) 18:252. doi: 10.1186/s13023-023-02858-4 37644448 PMC10466866

[37] Origa R Galanello R Ganz T Giagu N Maccioni L Faa G . Liver iron concentrations and urinary hepcidin in beta-thalassemia. Haematologica. (2007) 92:583–8. doi: 10.3324/haematol.10842 17488680

[38] Roghi A Cappellini MD Wood JC Musallam KM Patrizia P Fasulo MR . Absence of cardiac siderosis despite hepatic iron overload in Italian patients with thalassemia intermedia: an MRI T2^*^ study. Ann Hematol. (2010) 89:585–9. doi: 10.1007/s00277-009-0879-3 20016898

[39] Musallam KM Cappellini MD Wood JC Taher AT. Iron overload in non-transfusion-dependent thalassemia: a clinical perspective. Blood Rev. (2012) 26:16–9. doi: 10.1016/S0268-960X(12)70006-1 22631036

[40] Roberts AS Shetty AS Mellnick VM Pickhardt PJ Bhalla S Menias CO. Extramedullary haematopoiesis: radiological imaging features. Clin Radiol. (2016) 71:807–14. doi: 10.1016/j.crad.2016.05.014 27377325

[41] Huang Y Liu R Wei X Liu J Pan L Yang G . Erythropoiesis and iron homeostasis in non-transfusion-dependent thalassemia patients with extramedullary hematopoiesis. Biomed Res Int. (2019) 2019:4504302. doi: 10.1155/2019/4504302 30834265 PMC6374788

[42] Rao E Kumar Chandraker S Misha Singh M Kumar R. Global distribution of β-thalassemia mutations: an update. Gene. (2024) 896:148022. doi: 10.1016/j.gene.2023.148022 38007159

[43] Zhou Y Cao Y Fang Z Huang K Yang M Pang G . Research on the clinical factors of cardiac iron deposition in children with beta-thalassemia major. Eur J Pediatr. (2024) 183:875–82. doi: 10.1007/s00431-023-05300-w 37938353 PMC10912130

